# High-Throughput Methods in Materials Science (Part I): A Review of Chemical and Physical Methods and Automated Sample Logistics

**DOI:** 10.3390/ma19132853

**Published:** 2026-07-03

**Authors:** Krzysztof M. Nowak, Robert E. Przekop

**Affiliations:** Center for Advanced Technologies, Adam Mickiewicz University in Poznan, ul. Uniwersytetu Poznańskiego 10, 61-614 Poznań, Poland; krznow5@amu.edu.pl

**Keywords:** high-throughput experimentation, machine-readable data, automated polymer synthesis, closed-loop discovery, digital material management

## Abstract

**Highlights:**

- Automated “data factory” is one of the solutions to the data starvation problem in AI-driven discovery of polymers and composites.
- Robotic platforms with in-line rheology produce thousands of standardized material variants annually at low cost.
- Continuous Material Management with physical tagging ensures real-time metadata mapping for machine-readable data.

**Abstract:**

Artificial intelligence (AI) and machine learning (ML) algorithms possess the capability to accelerate the design of novel materials; however, their advancement in materials science is severely hindered by a fundamental deficit of experimental data, commonly referred to as data starvation. Unlike solution-based chemistry, where high-throughput (HT) technologies are a well-established standard, the automated synthesis of solid materials—particularly polymers and multicomponent composites—poses an extreme engineering challenge. Furthermore, the traditional, manual research model is inherently flawed by human bias, notably the systematic non-publication of negative results, which deprives AI models of critical boundary information regarding the design space. This paper is the first in a three-part review series defining the architecture of a fully automated, unbiased “data factory” for closed-loop discovery. This section focuses on the physical foundations of the HT workflow: experimental planning, automated synthesis, and material management. Emphasis is placed on the paradigm shift from classical, discrete Design of Experiments (DoE) to the novel concept of Continuous Gradient DoE. It reviews how robotic platforms utilizing precise gravimetric and volumetric feeders, integrated with extruders and in-line capillary rheology, enable the seamless, high-throughput manufacturing of thermoplastics and composites. Moreover, an innovative approach to sample logistics is presented, redefining classical storage patterns through the implementation of Continuous Material Management. This encompasses direct physical tagging (e.g., inkjet marking on continuous filaments or films), spool-based transport systems, and precise, real-time metadata mapping. As demonstrated, the integration of these systems yields an order-of-magnitude increase in productivity (generating tens of thousands of novel material variants annually), a radical reduction in unit costs, and the production of terabytes of standardized, machine-readable data. Establishing this reliable hardware and analytical infrastructure represents the essential first step toward unlocking the full potential of artificial intelligence in advanced materials engineering.

## 1. Introduction

Artificial intelligence (AI) and machine learning (ML) algorithms have reached an unprecedented level of maturity in recent years, offering the potential to radically accelerate the design of new materials. However, in the field of materials science, these systems increasingly encounter a fundamental barrier: the phenomenon of data starvation. The ability of predictive models to chart new synthesis pathways is directly constrained by a critical lack of accessible, standardized, multidimensional, and mass-generated experimental datasets that are fully machine-readable.

**Figure 1:**
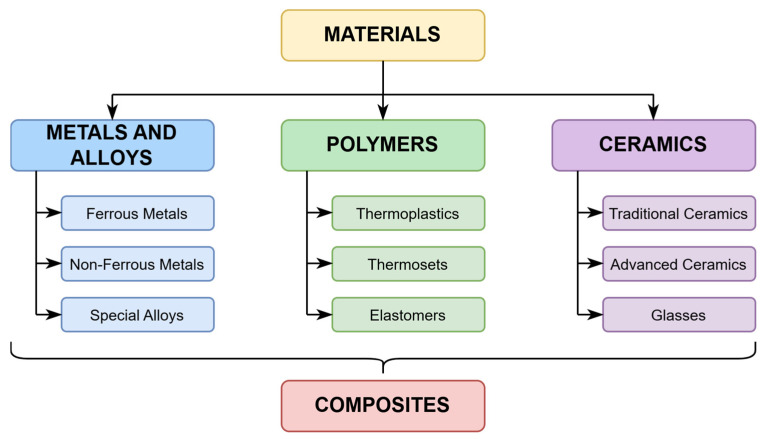
Classification of materials.

Understanding this deficit requires an examination of the evolution of laboratory methodologies. While high-throughput (HT) technologies—largely based on automated liquid handling—became the standard in chemical sciences and pharmacology decades ago, materials engineering faces severe hardware and technological bottlenecks in this regard. Automating the production of solid-state materials, including advanced thermoplastics, ceramics, and polymers with complex rheological properties, remains a massive engineering challenge. An even greater, almost entirely untapped niche is composite materials. The necessity to combine fundamentally different classes of materials and to strictly control rigorous processing regimes makes their high-throughput manufacturing exceedingly difficult. This technological barrier frequently exacerbates the strong conservatism within the scientific community. The adherence to traditional, manual, and artisanal approaches to creating individual samples results in unproportionally low productivity and actively slows down the transformation toward automated laboratories. The classification of materials into categories is shown in Figure 1.

Furthermore, this traditional workflow carries another highly destructive consequence for the development of AI: the systematic loss of knowledge. In the classical paradigm, the human researcher acts as a filter determining which data is disseminated. Negative results—such as failed syntheses, material degradation, or parameters falling below expectations—are widely marginalized and rarely published due to inherent publication bias and the pressure for academic success. However, from the perspective of machine learning algorithms, it is precisely this data on “failures” that defines the critical boundaries of the physicochemical design space. A lack of negative data inevitably leads to the training of distorted models that are incapable of broad generalization.

The implementation of automated High-Throughput methods is a promising path to break this impasse. Robotic HT platforms do not merely solve the throughput problem by accelerating experimental timelines; fundamentally, they serve as objective data factories, without taking the human factor into account. Every experiment, regardless of its outcome, is treated as a fully valid, rigorously tagged piece of information.

The potential impact of high-throughput automated platforms can be illustrated by the example of recently described autonomous laboratory systems. Burger et al. presented a robotic platform capable of performing 688 experiments over eight days, which corresponds to approximately 86 experiments per day and an estimated annual throughput exceeding 31,000 experiments under continuous operation [1]. Similarly, the Ada autonomous laboratory described by MacLeod et al. completed a full cycle of synthesis and characterization in about 20 min, allowing it to process approximately 72 samples per day, or over 26,000 samples per year. By comparison, conducting 1000 experiments using conventional, manual procedures can take several hundred working days, whereas autonomous systems are capable of performing comparable test series with minimal human intervention after initial configuration [2]. These platforms enable the generation of an order of magnitude more experimental data than traditional laboratory methods, which significantly increases the availability of machine-readable datasets essential for AI- and machine learning-based materials discovery. Furthermore, these systems enable the exploration of design spaces containing millions of possible parameter combinations, which would be practically impossible using conventional experimental methods [1,2].

Given the interdisciplinary complexity of this transition, this paper constitutes the first part of a three-part review series dedicated to the architecture of closed-loop materials discovery. This section focuses on the foundational, and arguably most challenging, stage: the physical execution, automation, and management of HT experiments in the demanding environment of solid-state and polymer materials. It provides a critical review of classical research approaches while highlighting the necessary paradigm shift from discrete Design of Experiments (DoE) to continuous, gradient-based methodologies. Furthermore, it discusses the engineering challenges of robotic synthesis and explores novel approaches to sample logistics, including continuous material management and real-time digital tagging. Having established these objectives, physical hardware foundations are a prerequisite for generating the massive volumes of high-quality data required to unlock the full predictive power of artificial intelligence in future materials engineering.

### 1.1. Motivation for This Review

In recent years, numerous review articles have addressed the increasingly rapid convergence of artificial intelligence, automation, and high-throughput experiments in the field of materials science. Early, visionary work focused on integrating robotics, computation, and data-driven approaches for autonomous material discovery, while more recent reviews have discussed self-driving laboratories, autonomous experimental systems, and machine learning-assisted research processes. This research has significantly contributed to the development of modern computational materials science and closed-loop optimization frameworks. Despite these advances, most existing reviews have focused primarily on artificial intelligence, machine learning algorithms, autonomous decision-making, or the general concept of autonomous laboratories. Relatively less attention has been paid to the physical and logistical infrastructure necessary to generate high-quality, machine-readable experimental data. To the best of the authors’ knowledge, no previous analysis has provided a systematic examination of the complete hardware architecture of high-throughput materials laboratories, including automated synthesis platforms, robotic handling systems, sample storage architectures, material tracking technologies, and FAIR-compliant metadata management. A comparison of other review articles is presented in Table 1. This analysis aims to fill this gap by focusing on the experimental and operational foundations necessary for the implementation of autonomous materials research environments [3,4,5,6].

### 1.2. Structure of the Review Series

Given the broad scope of high-throughput materials engineering, the review has been divided into three complementary parts addressing successive stages of autonomous materials discovery. Part I, presented in this manuscript, focuses on the physical and logistical foundations of high-throughput experimentation, including automated synthesis, material processing, robotic handling, sample identification, storage systems, and metadata management. Part II will concentrate on high-throughput characterization techniques, sensor integration, in situ and operando measurements, and methods of feature extraction from multimodal datasets. Finally, Part III will address data-driven materials engineering, covering artificial intelligence, machine learning, digital twins, autonomous optimization strategies, and the architecture of self-driving laboratories. Together, these three parts provide a comprehensive overview of the infrastructure required for closed-loop materials discovery platforms. Table 2 summarizes the structure of the review series.

## 2. Objective Definition and Design of Experiments

### 2.1. Objective Definition

The first step in planning a high-throughput experiment is to define the research objective and the corresponding scientific questions [7,8]. These objectives must be unambiguous and defined in such a way that they can be measured and evaluated with automated methods. When defining the objective for this type of process, it is essential to specify performance metrics that provide a quantitative description of the material under investigation. These metrics include individual parameters, composite indicators comprising multiple parameters, and multi-criteria functions that are the combination of criteria such as performance, stability and cost [8,9]. At this stage, it is crucial to identify the process constraints, which include the duration of the experiment, its cost and the availability of equipment. These constraints are significant in developing a workflow strategy and will determine the choice of methods during experiment planning [10].

The central element of the objective definition phase is the formal definition of experimental parameters, including variables relating to chemical composition, synthesis conditions (temperature, time, atmosphere), material architecture (number and thickness of layers, composition gradients), doping, and post-processing parameters [7,10]. Due to the wide range and high non-linearity of the parameters, at the research design stage, in a classical experimental approach, it is necessary to narrow down the range of parameter selection and identify those with the greatest application potential [11]. The use of high-throughput methods allows for an increase in the number of parameters studied [7,8].

The sources of knowledge for defining the research objective and scope include a literature review, data from preliminary simulations and computational models, and expert domain knowledge. The literature provides points of reference and identifies research gaps and under-explored areas; simulations allow for a preliminary assessment of trends and the elimination of areas with a low probability of success, whilst expert knowledge enables the formulation of hypotheses and the integration of all these elements, which, at an early stage of the research project, can significantly contribute to increasing the efficiency of subsequent stages of automated synthesis, characterization and data analysis [8,12].

### 2.2. Design of Experiments

Design of Experiments (DoE) is a formal statistical methodology used for systematic planning, execution, and analysis of experiments in order to determine which factors influence a given process or phenomenon. Its primary objective, particularly in high-throughput experimentation, is to maximize the amount of information obtained from a minimal number of measurements by efficiently exploring a multidimensional parameter space [13,14].

In classical DoE, the experimental space is discretized into a finite set of factor levels. Each experimental run corresponds to a specific combination of predefined levels of independent variables (factors), which may include compositional, processing, or environmental parameters. Core elements of DoE include factors, levels, responses, interactions, and experimental runs, which together define a structured sampling of the parameter space [13,14].

In contrast, Continuous Gradient DoE (CG-DoE) replaces the discrete sampling of factor levels with a quasi-continuous variation of one or more parameters across a spatial or temporal domain. Instead of conducting isolated experimental runs, CG-DoE implements gradients (e.g., compositional, thermal, pressure, or time-dependent gradients), where a continuum of experimental conditions is encoded within a single sample or experiment [15].

**Figure 2:**
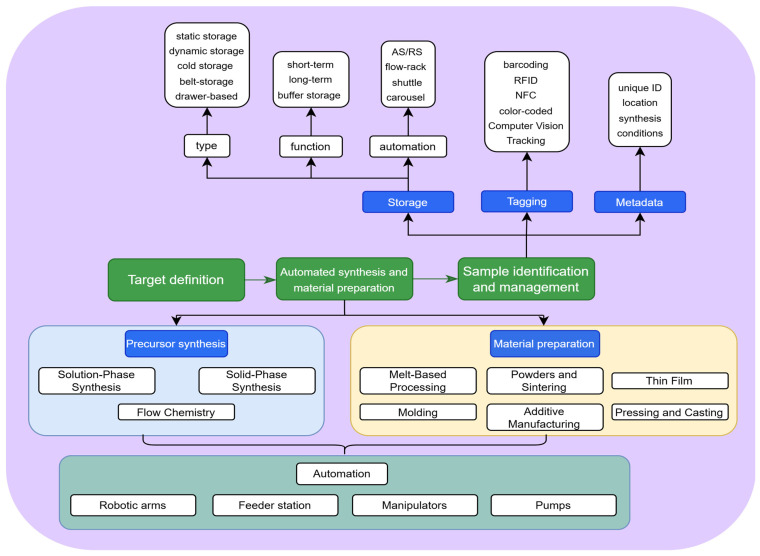
High-throughput experimentation workflow.

The fundamental distinction between classical DoE and CG-DoE lies in the representation and sampling of the design space: in classical DoE, the parameter space is sampled at discrete points defined by preselected levels. This allows straightforward statistical analysis and estimation of main effects and interactions but may require a large number of experiments for high-dimensional systems. In CG-DoE, the parameter space is sampled continuously along one or more gradients. As a result, a single experiment can encode a large number of effective conditions, significantly increasing throughput and resolution [15,16]. The workflow, DoE, and the methods used in it are summarized in Figure 2.

## 3. Automated Synthesis and Material Preparation

The automated synthesis of precursors and preparation of materials, utilizing artificial intelligence tools and robotics, is increasingly transforming modern materials engineering, enabling the rapid discovery of new compounds and the optimization of their properties. The selection of synthesis and sample preparation techniques is one of the key elements in designing and executing the workflow. It depends on factors such as the target material class, the required product form, the range of variability in composition and structure, and even the expected throughput of the HT experiment.

### 3.1. Precursor Synthesis

#### 3.1.1. Solution-Phase Synthesis

Solution-based methods in synthesis are among the most frequently automated techniques in organic chemistry, but they are increasingly being applied in materials synthesis as well [17]. These methods encompass a wide range of techniques, including sol–gel processing, co-precipitation, hydrothermal and solvothermal synthesis, as well as microvolume synthesis. The advantage of solution-based synthesis lies in its high flexibility with respect to modifying chemical composition, pH, reagent concentrations, and reaction conditions. At the same time, ensuring control over process reproducibility and kinetics remains challenging, requiring strict regulation of operational parameters and integration with monitoring systems [18,19]. One of the most widely used stationary systems is the Chemputer—a platform employing syringe pumps and multi-port valves to combine reagents, carry out phase separation, and perform filtration in predefined sequences. Equipped with all required hardware for executing reactions, the system allows full replication of manual laboratory procedures [17].

SynBot is another example of a platform designed for automated batch synthesis. It consists of three segments: artificial-intelligence-assisted retrosynthesis software, software controlling the robotic section, and the physical hardware itself, including dispensing modules, reaction stations, and analytical components. This architecture accelerates the discovery and optimization of chemical processes. Additionally, real-time monitoring improves synthesis quality and detects any deviations. The system enables efficient exploration of synthesis feasibility and material properties at early development stages while ensuring high quality and reproducibility [20].

AutoSyn is an example of a flow-synthesis platform designed for drug manufacturing at scales ranging from milligrams to grams. Owing to its digital standard for synthesis protocols, the platform ensures reproducibility and transferability of procedures. The process is managed using the CityScape architecture, which defines three operational layers: the assembly layer, the substrate layer, and the collector layer. The system is equipped with 16 pumps that enable seven continuous synthesis stages and two liquid–liquid separation processes. With three-level process planning and an integrated analytical platform, it is possible to generate up to 3887 unique flow pathways. According to the authors, the AutoSyn platform—together with its associated reaction databases—enables the synthesis of 87% of FDA-approved small-molecule drugs [21].

Another flow-synthesis platform is AlphaFlow, which integrates microfluidic channels with dedicated control software. The system is based on reinforcement learning within a single-period horizon, and its operational strategy relies on Ensemble Neural Networks. The authors report that AlphaFlow can efficiently navigate material synthesis design spaces even in the complete absence of prior information, outperforming other established optimization algorithms in this regard [22]. Table 3 summarizes the solution-based synthesis methods described in this section.

#### 3.1.2. Solid-Phase Synthesis

Solid-state synthesis from powders is a key part of producing materials for catalysis, ceramics, and electrode materials. The automation used in solid-state synthesis relies on robotic dosing, mixing, and sintering. Systems based on gravity feeding with continuous weighing and automated mixing mills are employed. In the case of ZrO_2_–Y_2_O_3_ material, the weighed and mixed powder is transferred to dies, where, after compaction, the materials are sintered in furnaces [23]. A key challenge in HT powder synthesis is ensuring compositional homogeneity and controlling particle size distribution. However, automated platforms allow for systematic modification of parameters such as grinding time, calcination temperature, and atmosphere, enabling the rapid identification of optimal synthesis conditions [7,8].

#### 3.1.3. Parallel Reactors

Parallel reactors constitute a key component of the experimental infrastructure in high-throughput systems, enabling the simultaneous execution of a large number of syntheses or material reactions under strictly controlled yet diverse processing conditions. This technique allows multiple reactions to be carried out at once, which significantly accelerates material screening in the context of HT workflows [24]. Reactions can be performed in several configurations, among which the most commonly used are as follows:

- Microfluidic flow reactors—systems in which reactions occur under controlled flow and well-defined reaction conditions [19];
- Batch reactor arrays—sets of reactors, vials, or autoclaves that enable reactions to be performed under different conditions and reagent ratios;
- High-pressure and high-temperature reactors—similar to batch reactor arrays, but designed for the synthesis of materials that require extreme processing environments;
- Gas-phase reactors—used for syntheses that require individually controlled atmospheric composition for each reaction channel [24,25].

### 3.2. Material Preparation

#### 3.2.1. Melt-Based Processing

Induction melting is based on the generation of eddy currents in a conductive material using an alternating electromagnetic field, which causes it to heat up and melt. Automation in this process is based on PLCs and SCADA systems that collect real-time data on energy consumption and process parameters [26].

This process is relatively well suited to automation for the following reasons:

- The heating power can be precisely controlled electronically [27];
- Integration with temperature sensors (e.g., pyrometers) is possible;
- The process can be coupled with model-based and AI control systems.

In modern systems, approaches based on digital twins, which are virtual representations of physical furnaces, and reinforcement learning methods are being developed, which enable dynamic regulation of the power of induction coils in real time, minimizing overheating and temperature inhomogeneities [27,28].

Arc melting, including vacuum arc remelting (VAR), is widely used for the production of high-purity alloys (e.g., Ti, Ni superalloys) [29,30]. The process involves generating an electric arc between an electrode and the material, causing it to melt in a controlled atmosphere or vacuum [31]. The automation of this process is developing particularly in the context of the integration of multiple devices via control systems (e.g., ROS2) and the automatic execution of melting sequences and the mixing of components [32]. The limitations of the process stem from its highly non-linear dynamics and the difficulty in maintaining the stability of the molten metal. In practice, whilst operational automation like sequencing or handling is possible, full automation of alloy quality control remains limited [31].

Automated welding is a widely used method of material preparation, enabling the repeatable fusion, joining and forming of workpieces in a fully automated manner. Modern systems utilize welding robots, multi-axis manipulators, vision sensors and sets of digitally controlled parameters, which allow for precise control of the molten metal pool and automatic tracking of programmed trajectories. For the monitoring and control of the process, AI, adaptive algorithms and digital twins are used. Thanks to them, it is possible for continuous correction of the process parameters, which increases the repeatability and quality of material [33,34,35,36]. These systems support automated process sequences—positioning, welding, cladding, and layer joining—making them useful in the preparation of preforms, test specimens and samples for materials testing. The use of vision systems and digital twins enables partial real-time quality control, although full automation remains limited due to the non-linear nature of the weld pool and the difficulty of accurately detecting defects during welding [35,37]. Automation in melt-based processes is summarized in Table 4.

#### 3.2.2. Powders and Sintering

##### Metals and Alloys

Metal sintering is rapidly evolving from a traditional thermal operation into a highly automated, high-throughput manufacturing stage, driven by the need for process stability, repeatability, and scalable mass production. Modern production facilities use semi-automated and fully automated furnace lines, including tunnel-type FAST/SPS systems equipped with pallet conveyors that move parts between sintering chambers and external cooling units. These solutions are designed to support higher throughput and continuous operation [38]. To boost throughput, manufacturers rely on stacked sintering configurations: vertical 1D stacks using graphite spacers, planar multi-die 2D arrangements, and combined 3D stacking assemblies. These approaches enable multiple components to be sintered simultaneously, which notably increases batch density. Robotic handling with machine vision is becoming a standard feature, providing precise pick-and-place operations for loading and unloading parts from fixtures. Automated systems generate accurate spatial coordinates to ensure consistent component positioning on carriers [38,39]. In laser-based metal sintering, in situ optical monitoring using photodiodes enables real-time detection of melt-pool instabilities, porosity, and other defects. Advanced sensor fusion approaches combining optical, thermal, and acoustic data have become foundational in process-quality monitoring [40]. However, advanced sintering techniques, such as FAST/SPS, predicting the precise flow of powder in molds with complex geometries still often rely on engineering intuition rather than fully automated predictive algorithms [38].

##### Ceramics

Spray drying is a widely used method to produce spherical granules with controlled size distribution that involves atomizing a liquid (solution or suspension) into fine droplets, which are then instantly dried in a stream of hot gas [41]. Spray drying processes increasingly rely on in-line sensing and DCS-based monitoring, with partial automation achieved through closed-loop moisture control [42]. While industrial systems still require operator input, laboratory-scale robotic platforms demonstrate growing potential for fully automated, contamination-free processing [43]. Key limitations in spray drying automation include the lack of real-time control of the particle size distribution, continued reliance on operator-driven optimization, and insufficient performance of conventional control models in highly nonlinear thermal systems [42].

In other granulation methods like wet granulation, automation has advanced significantly thanks to CFD-DEM modeling and digital twin systems, which enable predictive control in fluidized beds and spray granulators, supporting automatic regulation of air flow, droplet size and temperature [44]. Nonetheless, ensuring fully reliable control remains a difficult task due to the complex, turbulent interactions between gas and solids, and the rapidly changing growth dynamics in a wet environment. In dry granulation, the roller compaction process is well supported by PAT tools (NIR, microwave resonance, imaging), which enable automatic monitoring of web density and granule properties and allow for closed-loop control of roller pressure and speed [45]. Other issues include material-dependent variability and unpredictable defects, such as material hardening during processing or loss of tableting ability [46].

Ball milling is a core mechanochemical technique in which grinding media induce high-energy collisions, enabling particle size reduction, alloying, amorphization, or solid-state reactions. It is widely used in materials science and metallurgy due to its simplicity, scalability, and ability to drive reactions without solvents [47]. Modern automation focuses on three areas: performance optimization, remote monitoring, and high-throughput sample handling. Controller-optimizers adjust feed rates to keep the mill at ~80–95% load, using piezo-accelerometers to correlate drum vibration with fill level [48]. IoT-enabled systems allow remote supervision of motor temperature, humidity, and current draw without the need for physical contact with apparatus. High-throughput workflows automate reagent dosing and parallel milling, while safety modules detect blockage or overheating [49]. Despite progress in digitalized milling workflows, ball-milling automation still faces several technical barriers. The primary challenge is the lack of direct, real-time measurements inside the drum, forcing systems to infer load and milling efficiency indirectly and historically leading operators to under-load mills to avoid failures. Material variability—changes in hardness, moisture, or composition—requires constant self-tuning of control algorithms, complicating stable automation [48,50]. Wireless connectivity is another obstacle: steel milling vessels act as Faraday cages, preventing Wi-Fi/Bluetooth transmission unless special signal-permeable front panels are used [51]. Finally, mechanical wear remains a major concern: automated systems must detect belt loosening, fractures, or abnormal vibration patterns, while high-throughput multi-station mills often struggle to supply sufficient impact energy for highly non-linear mechanochemical reactions [52]. Power-based methods for ceramics are summarized in Table 5.

Sintering is a process of producing and processing materials that involves the consolidation and densification of powdered substances (metals, ceramics) under the influence of heat and/or pressure, without bringing the material to a state of complete melting [53]. Modern sintering processes increasingly rely on advanced in situ monitoring and control systems. Programmable logic controllers (PLC) and SCADA architectures provide the foundation for fully automated, sequential furnace control used in industrial powder metallurgy and large-scale ore sintering lines [54]. Automated uniaxial instruments such as sinterometers enable continuous tracking of punch displacement under constant load maintained by a hydraulic system, allowing precise kinetic analysis through real-time sintergrams [55]. In selective laser sintering (SLS), infrared thermal cameras map powder-bed temperature before scanning, enabling automatic laser-power correction to minimize thermal gradients and ensure uniform melting [56]. Ultrafast High-Temperature Sintering (UHS) uses Joule-heated carbon strips to achieve ceramic consolidation in ~10 s, offering a highly repeatable, automation-friendly platform suitable for high-throughput materials research [57].

Despite remarkable progress in modern sintering technologies, full autonomy at industrial scale remains limited. Processes such as iron-ore sintering still rely heavily on operator expertise, even when advisory systems are available. Nonlinear, strongly coupled process dynamics hinder accurate predictive modeling, often forcing the use of fuzzy-logic controllers that require large historical datasets and are complex to implement [53]. Hardware constraints also pose limits—for example, some laser-sintering controllers do not allow independent, simultaneous modulation of laser power and scanning motion, restricting continuous closed-loop control. Finally, material specificity remains a barrier: advanced methods such as flash sintering only work for materials with suitable electrical properties, preventing broad applicability across all ceramic systems [56,57].

Hot pressing is a widely used technology in manufacturing ceramics, microelectronics and composites for bonding or powder consolidation [58]. It involves applying high temperature and pressure simultaneously to join parts or compact powder [59]. Current automation solutions focus on the precise control of physical parameters and the elimination of manual labor in challenging conditions. Modern systems employ microprocessor-based control of temperature, pressure, and dwell time to ensure fully repeatable cycles [58], while robotic manipulators handle loading and unloading of hot components, reducing manual work in hazardous environments [60]. Machine-vision systems (e.g., CCD cameras) assist with automatic alignment and calibration of parts before pressing, improving precision for variable geometries [58]. Advanced control algorithms, including fuzzy logic and adaptive schemes such as Dynamic Surface Control, compensate for nonlinear hydraulic behavior and temperature-dependent changes in material response [61]. Industrial presses also integrate automated lubrication via programmed spray systems, removing the need for manual graphite application [60]. Modern automation faces challenges arising from technological barriers. The extreme operating environment—with temperatures up to 1100–1200 °C, high compressive forces, and pervasive graphite dust—rapidly degrades sensors, robotic grippers, and vision systems, limiting long-term reliability. Real-time precision is also difficult to maintain because hydraulic system parameters drift significantly under heat, complicating thickness and pressure control for materials [58,61].

In the field of pressing, a key trend is the full automation of operations related to the preparation of ceramic mixtures. High-throughput powder dosing systems allow for the simultaneous formation of up to 100 samples with very high precision (up to 0.005 g), which accelerates material experiments and creates the databases necessary for ML methods. Such platforms meet the needs of high-throughput screening, enabling rapid prototyping and testing of numerous ceramic compositions [62,63]. Pressure-assisted slip casting significantly boosts production efficiency and is widely used in industry, enabling partial automation through controlled pressure and fluid flow. Furthermore, pressure die casting has been deemed more suitable for automation than conventional methods due to its faster process and compatibility with engineering molds [64,65].

The methods described above have been compiled and summarized in Table 6.

#### 3.2.3. Thin Films

Thin-film techniques are used in fields such as photovoltaics, electronics, surface catalysis, and sensing. They enable precise control over chemical composition, thickness, microstructure, and interfacial properties—crucial factors in the development of functional materials. Sputtering is one of the thin-film techniques that can be fully automated. Modern systems employ controllers based on Bayesian optimization algorithms, which adjust power supply parameters in real time using optical emission spectroscopy (OES) data to achieve the desired film composition [66].

In Chemical Vapor Deposition (CVD), a thin film is produced by chemical reactions of gaseous precursors at or near the substrate surface. This method is widely used for synthesizing semiconductors and carbon-based materials. It is routinely applied to the growth of carbon nanotubes, where automated platforms use Raman spectroscopy for real-time monitoring of the synthesis process [67].

Another automated thin-film method is Pulsed Laser Deposition (PLD), based on the ablation of a target material using high-energy laser pulses. This generates a plasma plume that transfers material onto the substrate. PLD enables atomic-level control of film growth, facilitating the creation of artificial crystal lattices and heterostructures [67,68]. Recently, PLD has gained importance in automated closed-loop systems [17,69]. The method has been used, for example, to produce TiO_2_ layers with thicknesses from 0 to 40 nm on conductive substrates. In high-throughput platforms, thin-film techniques are frequently implemented in a combinatorial mode, enabling the simultaneous fabrication of material libraries featuring gradients in composition or processing parameters [70].

#### 3.2.4. Molding

Molding encompasses a wide range of manufacturing processes used to give materials—most commonly polymers—specific shapes through the application of heat, pressure and controlled flow within a molding system. These techniques are of fundamental importance to modern manufacturing due to their scalability, repeatability and ability to produce complex components with high precision. Several key methods are widely used in this category, each differing in process mechanics, tooling requirements and scope of application. The best-known methods include extrusion, injection molding, blow molding and thermoforming. Together, they form the basic technological framework for transforming polymer raw materials into functional products across various industries, from packaging to the automotive sector and consumer goods [71,72,73,74].

##### Polymers

The extrusion process involves heating raw polymers until they melt, and then forcing this mass through a die (mold) under pressure generated by a screw [75]. As the material is transported through the cylinder, the screw mixes and kneads it, resulting in a homogeneous melt ready for shaping [76]. The final product, with a constant cross-section, emerges from the die in a continuous stream, after which it is cooled to achieve its final form, such as a pipe or film [75]. Polymer extrusion is a process that is currently undergoing a transformation towards ‘smart’ solutions and Industry 4.0. The integration of advanced control systems with physical modeling enables the optimization of product quality whilst reducing waste [76,77]. Modern polymer-extrusion lines increasingly employ intelligent pressure and melt-stability control, where neuro-fuzzy ANFIS regulators dynamically adjust screw speed to maintain target melt pressure and suppress surging, improving dimensional stability of extruded material [78]. Modern extrusion systems employ end-to-end process optimization, using Model Predictive Control (MPC) together with multi-task learning to coordinate not only the extruder itself but also downstream operations such as cooling, sizing, and cutting. These systems can forecast process trajectories up to an hour ahead [79]. Because direct melt-viscosity measurement is impractical at industrial scales, extrusion lines rely on soft-sensor viscosity estimation derived from torque, die-pressure, and temperature signals [78,80]. In pharmaceutical extrusion (HME) and advanced polymer processing, spectroscopy like NIR, Raman and UV-VIS provides real-time monitoring of chemical composition, drug load, and solid-state transitions without stopping the process [81]. High-throughput extrusion workflows make use of automated micro-extruders capable of generating compositional libraries with rapid, automatic changes in formulation every 20–45 s, supporting fast materials screening [82]. Industrial lines also integrate automatic physical-parameter control, including recipe switching, real-time width and thickness regulation for sheet production, and automated defect sorting to maintain consistent product quality. Polymer-extrusion automation is still constrained by several nonlinear and operational complexities. The process exhibits strong nonlinear behavior and long transport delays—for example, a change in screw speed may influence product dimensions only minutes later—making classical PID control insufficient [79]. Multiphase modeling is also difficult: flows containing fibers or particulate fillers are affected by wall-slip, fiber breakage, and shear-dependent viscosity shifts, complicating predictive control [74]. Many critical quality attributes, such as molecular weight or polymer degradation, remain inaccessible to real-time in-line measurement outside laboratory environments. Finally, AI deployment is hindered by data scarcity in industrial settings and the shortage of specialists who can bridge polymer-processing expertise with data-science skills [75].

Injection molding and blow molding are a cyclic manufacturing technologies used to produce highly precise thermoplastic components. Today, this field is undergoing a transition toward Industry 4.0, where intelligent, data-driven automation replaces manual tuning and operator experience. Modern molding systems integrate advanced sensing, AI-based control, and digital connectivity, making the processes increasingly autonomous, stable, and predictable [83,84].

A central area of progress involves AI-enabled temperature and process control. In blow-molding systems—especially stretch-blow molding—deep reinforcement learning algorithms are used to automatically adjust the IR-heating profile of preforms. These models compensate for environmental temperature fluctuations, uneven lamp aging, and process drift, stabilizing wall thickness and improving bottle consistency [85]. Similar approaches in injection molding use neural networks or genetic algorithms to optimize cycle parameters, reducing defects such as shrinkage and warpage [83]. Injection molding has also benefited from automated V/P switchover (velocity-to-pressure switchover). Moment of switch from filling to packing control—traditionally operator-dependent operation—can now be determined using pressure-gradient detection, machine vibration signatures, or ultrasonic sensors. As a result, the weight consistency of the parts is significantly improved [84,86]. Another major advancement is multimodal in-line quality control. Instead of relying on manual inspection or periodic sampling, real-time monitoring systems now fuse data from in-mold pressure and temperature sensors with electronic weighing, 3D metrology, and machine-vision tools powered by convolutional neural networks [84,87]. This enables 100% part-by-part inspection without extending the cycle time. Automated systems can detect deviations in geometry, surface defects, and weight variability immediately, allowing instant corrective adjustments [88]. Automation also addresses one of the challenges in polymer processing: variations in melt viscosity. Closed-loop “auto-viscosity” systems infer viscosity changes from nozzle pressure, cavity pressure, and torque measurements. They then adjust injection speed, screw plasticizing conditions, or hold pressure in real time. This is particularly important for recycled polymers where batch-to-batch inconsistency is unavoidable [89,90]. A strategic development supporting automation is the adoption of digital twins and CAE-enhanced control. Digital twins combine simulation models with live machine data to represent the real production environment continuously. They predict defects such as weld lines, sink marks, or air traps and help determine optimal first-shot parameters, reducing the need for manual trial-and-error during machine setup. The integration of CAE simulations into machine controllers improves startup efficiency and enhances process understanding [84,88]. The automation extends into tooling and mechanical hardware as well. Modern molds are equipped with synchronized slides, lifters, collapsing cores, or rotating inserts, all of which operate under coordinated electronic control. Even additively manufactured mold inserts can incorporate automated mechanisms, enabling complex geometries and undercuts without manual intervention [91].

A number of design and operational challenges continue to limit the full automation of molding processes, even with the use of modern Industry 4.0 technologies. One of the most difficult problems is the variability of recycled raw materials: polymers from post-consumer or post-industrial recycling streams exhibit unpredictable fluctuations in molecular weight and rheological properties. These variations directly affect flow, pressure stability, and thermal equilibrium inside the mold, making it difficult for control systems to maintain a constant range of processing parameters [89]. Another limiting factor is sensor technology. The conditions inside the mold—high pressure, elevated temperatures, and abrasive fillers lead to accelerated wear, calibration drift, and frequent sensor failures. Replacement and maintenance costs remain high, and long-term durability is still insufficient in many industrial environments [86,91]. The automation of the polymer molding process described above is summarized in Table 7. 

#### 3.2.5. Additive Manufacturing

##### Metals and Alloys

Additive manufacturing (AM) of metals, which includes processes such as powder bed fusion (PBF) and direct energy deposition (DED), is currently undergoing a transformation toward full automation and Industry 4.0 [92,93]. Given the high value of the components produced, the key objective is to eliminate process errors in real time [94]. Modern metal additive manufacturing systems increasingly rely on multimodal in situ monitoring, combining high-speed optical cameras, photodiodes for optical-emission analysis, acoustic sensors, and infrared or thermographic imaging. This fusion of visual, thermal, acoustic, and optical signals provides a detailed, real-time picture of melt-pool dynamics and powder-bed quality, enabling early detection of instabilities and defects [93,95,96]. To interpret data streams effectively, manufacturers are deploying machine-learning and AI models capable of identifying porosity, cracking, spatter anomalies, and insufficient fusion with accuracies frequently exceeding 90%. These models also support automatic tuning of process parameters—such as laser power, scan speed, or beam focus—based on predicted defect likelihood [92,96,97]. Such monitoring advances lay the groundwork for closed-loop control, in which LPBF or EBM machines dynamically adjust energy input during the build. Real-time feedback from cameras, pyrometers, or photodiodes allows the system to compensate for hotspots, prevent lack-of-fusion regions, and stabilize melt-pool geometry layer by layer [93,98]. In deposition-based processes like DED or WAAM, automation extends to robotic path planning, where multi-axis industrial robots compute conformal deposition paths over complex geometries. This supports uniform bead formation on curved or multi-orientation surfaces and reduces manual programming effort [99]. Another key development is the adoption of digital twins, virtual counterparts of physical AM machines that continuously ingest sensor data and simulate upcoming process states. These models enable predictive correction of defects, tuning of scan strategies, and optimization of deposition parameters—substantially improving repeatability across builds [94].

Metal additive manufacturing still faces several obstacles that limit full automation. The melt-pool physics is inherently chaotic, which makes it difficult to obtain consistent microstructures or repeatable material properties between builds [94]. At the same time, multimodal monitoring generates huge volumes of sensor data that must be processed in real time, requiring advanced edge-computing capabilities [92]. Sensors also face harsh operating conditions—high temperatures, vacuum environments in EBM, and constant exposure to powder and spatter—which reduces their durability and measurement accuracy [100]. A major systemic limitation is the absence of universal standards for data fusion, monitoring methods and quality assurance, meaning that solutions developed for one machine or alloy rarely transfer to another [101,102]. Data-driven approaches are additionally constrained by imbalanced datasets: defects are rare compared to normal layers, which makes it harder for AI models to learn robust failure-prediction patterns [92]. Despite progress, human intervention remains necessary, especially for tuning toolpaths or temperature-related parameters between layers [101]. A summary of automation strategies in metal additive manufacturing is presented in Table 8.

##### Polymers

Additive manufacturing of polymers, which includes techniques such as FFF, SLA, DLP, and SLS, forms the foundation of modern rapid prototyping and on-demand manufacturing and is increasingly being integrated into automation systems [103].

Fused Filament Fabrication is a polymer 3D-printing process in which a thermoplastic filament is fed through a heated nozzle and deposited layer-by-layer onto a build platform to form the final part [104]. Modern FFF systems increasingly incorporate in situ monitoring, using accelerometers to observe vibration patterns, acoustic-emission sensors to detect cracking events, and infrared cameras to track the real-time thermal distribution of the print [105,106]. Machine-learning algorithms—most commonly artificial neural networks and Random Forest models—are applied to automatically optimize print parameters such as speed, temperature, and layer height, improving strength and surface finish. Additionally, closed-loop vision-based systems can detect extrusion errors during printing and adjust deposition parameters in real time [105,107]. Research groups also develop robotized experimental platforms, where fully automated setups print samples (often from pellet-fed extruders), transfer them via robotic manipulators, and perform mechanical tests without human involvement [108,109]. Despite growing automation, FFF still suffers from performance limitations. Mechanical anisotropy and poor surface finish remain inherent drawbacks compared to subtractive or powder-based processes [104,110]. Also, mechanical failures—such as nozzle clogging or filament slipping—are frequent and difficult to predict without more advanced physical models [106].

SLA and DLP are vat-photopolymerization technologies in which UV light selectively cures liquid resin. SLA uses a scanning laser to draw each layer point-by-point, while DLP employs a projector to expose an entire layer simultaneously, enabling substantially faster build rates [104,111,112]. Automation in SLA/DLP focuses heavily on data-driven prediction and materials optimization. Neural-network models are used to forecast mechanical properties—such as hardness and surface roughness—based on exposure settings and resin parameters [105,107]. In advanced self-driving labs, AI systems automate the formulation of resin blends, tailoring photopolymer chemistry to desired functional requirements without manual intervention [108]. Despite these advances, SLA and DLP technologies still face limitations, the most significant of which is the need for post-processing, which is difficult to fully automate in a clean and efficient manner [104,108].

Selective Laser Sintering is a powder-bed fusion process in which a laser sinters layers of polymer powder inside a heated chamber with tightly controlled thermal conditions. Modern SLS systems incorporate in situ monitoring, tracking powder-bed temperature using multimodal sensors combined with deep-learning models capable of detecting layer-level defects [110,111,112]. Automated post-processing lines now handle powder removal (decaking) with robotic systems, improving operator safety and accelerating material recycling [113].

A summary of automation strategies in polymer additive manufacturing is presented in Table 9.

### 3.3. Automation

Automation of laboratory processes transforms traditional organic and inorganic synthesis into a machine-controlled workflow, allowing human resources to be freed from tedious and repetitive tasks while accelerating the discovery of new materials. A key aspect of modern automated platforms is the integration of hardware with advanced software and synthesis-planning algorithms. Beyond precursor synthesis, automation plays an equally crucial role in material preparation and post-synthetic processing, which vastly expands the functional capabilities of high-throughput research environments. Such integration is essential for implementing closed-loop experimental workflows, in which synthesis and characterization results are used in real time to plan subsequent iterations of the investigation. In this context, automation becomes not only a means of increasing throughput but also a central component of data-driven materials discovery [114,115].

#### 3.3.1. Robotic Arms

Robotic arms in automated workstations act as connectors between individual modules and are used for the precise transfer of samples between synthesis, preparation, and characterization units, as well as for performing repetitive operations such as loading and unloading reactors, manipulating thin-film substrates, or handling multiwell plates. These arms may be mounted on fixed platforms or linear rails, significantly enhancing their working range and flexibility within the laboratory [116]. Their use enables continuous operation, supporting high-throughput execution of synthesis protocols [116,117]. Industrial robotics offer exceptionally high repeatability and positioning precision, often in the range of ±0.01 mm to ±0.1 mm, which is crucial for achieving reproducible experimental results [118,119].

#### 3.3.2. Feeding Stations

Precise dosing of substrates is fundamental for reproducible synthesis. Feeding stations are responsible for the automated delivery of materials, reagents, and components. For dispensing solids, automatic weighing systems are employed to accurately measure the required quantities of powders [117]. Loss-in-weight feeders controlled by PLC systems are widely used, ensuring a stable and precise flow of granular materials [120]. Liquid dispensing automation relies on robotic pipetting systems or precision pumps, often implemented in multichannel configurations capable of supporting thousands of small-scale reactions [116,117].

In designing a dispensing platform, both quantitative accuracy and full traceability of supplied resources are considered, often achieved through integration with sample-coding systems and laboratory databases. As a result, feeder stations support process standardization and a reduction in experimental variability.

#### 3.3.3. Manipulators

Manipulators are a specialized class of devices designed to perform precise and complex operations on samples or laboratory components [121]. Unlike robotic arms, manipulators are typically dedicated to specific tasks or function as end-effectors mounted on robotic arms. They play a crucial role in processes requiring high accuracy and stability, such as thin-film deposition, precision mixing of reagents, or positioning samples for in situ characterization. Manipulators are engineered with a structure analogous to human anatomy, incorporating “joint-like” elements, and a key feature is the ability to rapidly exchange end-effectors. This allows a single manipulator to function as a container gripper, dosing tool, or even a mechanical grinder for reducing synthesized products to fine powders [122,123,124,125].

Table 10 below summarizes the key automation components in robotic laboratory workflows.

## 4. Sample Identification and Management

### 4.1. Storage

#### 4.1.1. Type of Storage

##### Static Storage

In this type of storage facility, samples are kept in fixed, non-moving locations, such as on shelves or in cassettes. Such systems are based on simple architecture, but this is accompanied by high reliability and an easy implementation process. As a result, this type of solution is attractive at the early stages of automation [126,127]. In high-throughput processes, static storage is used for the long-term storage of reference or archive samples. An example of a static storage system is one comprising static pallet racks with wide or narrow aisles (APR/NAPR) [128].

##### Dynamic Storage

In dynamic storage systems, samples are actively moved within the storage areas to optimize access to them or space utilization, with the ultimate aim of increasing throughput. In dynamic storage, the allocation of space for a specific sample takes place in real time, thereby preventing the system from becoming blocked and optimizing space. Movement within the warehouse is usually gravity-driven via appropriately sloped tracks or by external forces such as pneumatic, hydraulic or electric motors. Integration with control systems allows for the adaptive reorganization of the warehouse according to current experimental needs, which is notably important in closed-loop research systems [127].

##### Cold Storage

Cold storage refers to facilities that provide the appropriate temperature conditions required for samples that are chemically, biologically or physically sensitive. This type of storage includes cold rooms and freezers, the design of which requires high thermal insulation and specialized substrates to protect against deformation caused by low temperatures. Advanced cold storage facilities utilize technologies such as LHCS (Latent Heat Cold Storage) systems, which employ phase-change materials to store thermal energy, whilst sample storage takes place in a controlled atmosphere with regulated levels of oxygen and carbon dioxide [128,129]. In high-throughput environments, cold storage must combine precise temperature control with high operational repeatability and minimize the time samples are exposed to external conditions. These systems are important in research on electrolytes, hybrid materials, functional inks and samples intended for long-term analysis [130].

##### Belt-Based Storage

Belt-type systems are belt- or conveyor-based storage systems used for the short-term storage or transport of samples between successive stages of a planned workflow. These systems are common in production lines but are increasingly being adapted for high-throughput platforms where a continuous flow of samples is required. In parallel storage systems (PSS), horizontal conveyors combined with vertical screw conveyors allow for the parallel and rapid retrieval of products from multiple rack levels simultaneously. Belt conveyors are often integrated with sorting robots and AGVs, creating automated transport lines within the warehouse. The entire process is usually controlled by PLCs, which allow for real-time control of the speed and direction of sample movement [131,132,133]. The advantage of belt-based storage is its high throughput and the ability to integrate seamlessly with synthesis, characterization and sorting modules. A limitation, however, is the reduced flexibility in random access to individual samples, which means that these systems are most often used as intermediate elements rather than long-term storage facilities.

##### Drawer-Based Storage

Drawer-based storage systems are based on modular cabinets or cassettes that can be pulled out independently and operated automatically by robotic arms or manipulators. The use of this system ensures high storage density with rapid access, creating a compromise that is ideally suited to high-throughput platforms. A common application of drawer-based storage in the pharmaceutical industry is in automated medicine dispensing cabinets, where this solution minimizes the risk of incorrect medicine dispensing [134]. In high-throughput platforms, drawer-based storage is frequently used for storing thin-film substrates or reaction vials. The modular design allows for easy scaling of the system and its reconfiguration to meet changing research needs. Modern systems such as TansuBot use mobile cameras to monitor the contents of the storage facility, which, in turn, facilitates the retrieval of the required product based on images or sample history [135].

Types of storages described above are collected and compared in Table 11.

#### 4.1.2. Function of Storage

The storage types mentioned above can serve different functions. In automated high-throughput platforms, the function of sample storage extends far beyond passive containment. Storage systems act as an active segment of the workflow, managing sample flow, ensuring material stability, and synchronizing individual process stages. Based on their function, storage systems can be divided into three categories.

The first category consists of short-term storage. Short-term storage includes systems designed for the temporary holding of samples during a single experimental process. Typically, these systems contain materials with high rotation between stations and short shelf life. Samples stored in such systems are usually positioned between consecutive stages of synthesis, processing, or characterization. Short-term storage must ensure rapid and consistent access to samples, minimizing operational delays and enabling smooth execution of planned experimental sequences. For this reason, these storage units are usually located close to working modules and optimized for high throughput as well as integration with robotic arms and manipulators.

The second category, long-term storage, serves the opposite function. These systems are intended for storing resources over extended periods, often for archiving, maintaining reference samples, or enabling later distribution. In long-term storage, maintaining stable conditions for the entire storage duration is crucial to prevent any changes in the stored samples. Such systems are typically designed with high sample-density capacity and reliability in mind, even at the expense of longer access times.

The third category is buffer storage. These systems serve as intermediaries between synthesis and production modules, and sample preparation and characterization stations. Buffer units balance differences in production rates, supply and demand, and the varying durations of individual process stages. As a result, the platform can maintain high throughput even when different stages of the process operate at significantly different speeds or when delays occur in one of the stages. Buffer storage is typically highly automated and optimized for rapid sample transfer, often utilizing dynamic systems.

#### 4.1.3. Automation in Storage

The automation of storage systems must ensure rapid and repeatable access to samples, unambiguous traceability, and full integration with control and experimental planning systems. This makes automated storage a critical element of high-throughput infrastructure. In modern research platforms, sample storage units no longer function as passive repositories; instead, they become active workflow components capable of dynamically responding to experimental needs. The automation systems used in storage facilities are listed in Table 12 and described below.

Automated Storage and Retrieval Systems (AS/RS) are systems that incorporate fully automated, computer-controlled storage facilities operated by mechanical devices such as linear robots or multi-axis manipulators. AS/RS enables precise positioning of samples within the three-dimensional storage environment and their rapid delivery to other stations. In high-throughput processes, the use of AS/RS is particularly advantageous for large sample libraries that require dense packing and continuous identification [136,137,138].

Flow-rack systems are semi-automated storage units in which samples move along guides or roller tracks. These systems are often used for short-term storage and as buffer warehouses between sequential workflow stages. They provide rapid access to samples within a simple architectural layout, and integration with robotic arms allows for automatic retrieval and transfer, supporting smooth execution of high-throughput processes [139,140,141,142].

Automation in shuttle-based systems relies on autonomous carts or track-guided platforms that transport samples between storage locations and workstations. This type of automation is especially effective in large-scale storage environments where frequent access to randomly selected samples is required. Due to their modular architecture, shuttle-based systems can be scaled as the number of samples increases and adapted to evolving workflow demands [143,144,145].

Carousel-based systems utilize rotating storage structures in which samples are placed in containers mounted on a rotating frame. This design enables rapid delivery of a selected sample to the access point, minimizing waiting time. Such systems are commonly used as dynamic medium-capacity storage units, offering a favorable balance between access speed, packing density, and technical complexity [146,147].

### 4.2. Tagging

In environments where hundreds of samples are processed simultaneously, maintaining full sample identification is essential for the entire workflow. Unambiguous sample identification determines data consistency and enables complete tracing of each sample’s experimental history. With appropriate tagging, seamless transfer of samples between synthesis, storage, and characterization modules becomes possible. In practice, various tagging technologies are used in automated and high-throughput processes [148].

The most common identification method in automated laboratories—due to its simplicity, low cost, and high reliability—is barcoding. One-dimensional (1D) and two-dimensional (2D) barcodes can be easily applied to vials, sample cassettes, or multiwell plates. Particular attention is given to 2D codes such as QR, which allow storing larger amounts of information while maintaining a small tag size. QR codes can encode not only identification data but also process metadata or material-specific information. They are especially useful in systems requiring flexibility and easy access to information [149,150].

Barcoding must be integrated with vision systems or scanners to enable identifier reading during robotic operations. A key limitation of barcoding is its line-of-sight requirement, meaning that label readability depends on direct visual accessibility. Therefore, when choosing this tagging method, factors such as sample type, protective packaging, and potential mechanical or chemical damage to the label must be considered [151,152].

Tagging based on RFID (Radio-Frequency Identification) technology relies on wireless communication between an RFID tag attached to the sample and an RFID reader. This method enables identification without optical line-of-sight or physical contact, significantly improving operational reliability and flexibility [152,153]. RFID tags, depending on range, memory capacity, and power requirements, may be passive, active, or semi-active. Based on operating frequency, three categories of RFID are distinguished:

- Low Frequency (RFID LF)—125–134 kHz, short range (up to 10 cm), high resistance to interference, but increasingly uncommon;
- High Frequency (RFID HF)—13.56 MHz, moderate range (up to 1 m), good compatibility with liquids and dielectric materials;
- Ultra-High Frequency (RFID UHF)—860–960 MHz, long range (3–10 m) and capability to read multiple tags simultaneously [154,155].

In laboratory environments, small passive tags compatible with various sample carriers are most commonly used. RFID readers are often integrated with robotic arms, storage stations, or conveyor systems, enabling automatic tag detection during each manipulation step. A major advantage of RFID is the ability to read multiple samples simultaneously due to anti-collision protocols that coordinate sequential communication with individual tags without signal interference, thereby improving operational throughput [156,157].

RFID also enables real-time sample location tracking, which is particularly important in large, multimodular HT systems. Additionally, RFID tags can store auxiliary information such as processing history or storage conditions, increasing the autonomy of sample-management systems [158,159].

Another popular tagging method is NFC (Near-Field Communication), a variant of RFID operating at 13.56 MHz with a very short range (approximately 4–8 cm). NFC is mainly used in local and manual interactions. In high-throughput workflows, it is especially useful at interface points between automated and manual stages, enabling fast and intuitive access to sample information [160].

Computer Vision Tracking is a tagging-free method based on image-recognition algorithms, where sample identification and tracking rely on visual features such as shape, position, pattern, or other distinguishing characteristics [161]. These methods are particularly valuable when physical tagging is difficult or impossible, for example in the case of thin films or nanostructured materials [162]. When combined with color-coded marking, which uses color patterns or visual cues, these approaches constitute a fully passive, contact-free identification method well suited for highly automated environments with intensive mechanical handling [163].

Although color-coded tagging has low information capacity, it complements vision-based systems effectively. Color markings may take various forms, such as the following:

- Solid colors;
- Multicolor rings or bands;
- High-contrast geometric patterns;
- Combinations of colors and shapes [164].

The placement and design of visual markers must be engineered to withstand process conditions and remain compatible with existing robotic handling systems [165]. Modern computer-vision systems use advanced algorithms for image segmentation, feature extraction, and multi-object tracking. For high-throughput processes, it is crucial that these systems can detect many samples simultaneously and differentiate between visually similar objects [166]. However, computer vision and color-coded tagging do not provide globally unique identifiers, which limit their use as a standalone method. In laboratory practice, they are most often deployed as a complementary layer, operating alongside barcoding or RFID to increase robustness and redundancy of the identification system [167,168]. A comparison of sample identification and tracking technologies in automated laboratories is presented in Table 13.

### 4.3. Metadata

Metadata serve as the link between a physical sample and its digital representation within data-management systems. Autonomous and semi-autonomous high-throughput systems require complete, unambiguous, and machine-readable specifications of the information describing each sample at all stages of its lifecycle. Metadata not only enables unique sample identification but also determines experimental reproducibility, data consistency, and the ability to perform subsequent statistical analysis and modeling.

Following the FAIR principles, metadata management systems must ensure that metadata are

- Findable—each sample or dataset requires a persistent unique identifier that can be indexed and retrieved through searchable descriptors;
- Accessible—metadata should be stored in standardized, machine-readable formats and retrievable through defined protocols, even when raw data remain restricted;
- Interoperable—they should follow consistent schemas, vocabularies, and units to ensure compatibility across instruments, laboratories, and databases;
- Reusable—metadata must comprehensively document the experimental context, including synthesis conditions, instrument settings, calibration, timestamps, and data provenance [169,170,171].

The implementation of FAIR principles in materials science has led to the development of a range of metadata standards, data infrastructures, and domain-specific repositories aimed at improving data exchange and interoperability. General metadata frameworks, such as the Dublin Core Metadata Element Set, provide a minimal, standardized description of digital resources and define basic elements, including identifiers, creators, descriptions, and provenance information. However, due to the complexity of research processes in the field of materials science, more specialized approaches have been developed. The ISA-Tab framework enables a structured description of experimental research by linking information at the levels of experiments, analyses, and samples, making it particularly suitable for complex laboratory processes involving multiple experimental stages [172,173].

Several specialized initiatives have emerged in the field of computational and data-driven materials science. The Materials Genome Initiative (MGI) introduced the concept of an integrated materials data infrastructure, emphasizing the standardization of data representation, data sharing, and interoperability between experimental and computational methods [174]. Within this framework, databases and platforms such as the NOMAD provide extensive repositories of materials data accompanied by structured metadata. NOMAD employs a schema-based metadata representation to harmonize heterogeneous computational results and supports FAIR data principles, enabling the storage, retrieval, and reuse of materials data in a machine-readable format [175]. Similarly, the OPTIMADE standard defines a common application programming interface (API) for accessing distributed material databases, allowing different repositories to exchange information on structure and properties using a unified representation [176].

To achieve true interoperability between heterogeneous systems, metadata representation increasingly relies on formal ontologies and semantic data models. Ontologies define relationships between concepts, materials entities, processes, and measurements, allowing machines to interpret the meaning of stored information rather than only its structure. In materials science, ontology-based approaches support integration of experimental and computational data by providing common vocabularies and semantic relationships between datasets [177]. Machine-readable metadata formats, including JSON-LD (JavaScript Object Notation for Linked Data), enable the representation of such semantic information in a format that can be directly processed by computational systems and artificial intelligence algorithms. The combination of standardized schemas, ontologies, and machine-readable formats is therefore a key requirement for autonomous experimentation platforms, where metadata must be automatically generated, interpreted, and transferred between different laboratory modules [169,178].

In automated and HT environments, metadata collection should be embedded directly into workflows to minimize manual input and maintain consistency. Properly designed FAIR metadata systems enable scalable, reproducible, and data-driven research, forming the foundation for autonomous experimentation and digital materials design.

Depending on the type of metadata, they can be divided into several categories. The fundamental category is the unique identifier (UID) that allows a given sample to be distinguished unambiguously from all others within the system. The UID is the central point of reference to which all additional experimental information, measurement data, and operational changes are assigned [179,180]. Typically, the UID is generated automatically and linked to a physical sample tag such as a barcode or RFID [181]. The stability and immutability of the UID throughout the entire sample lifecycle are crucial for tracking its history and integrating data originating from different modules of the platform [169].

Another type is location metadata, which describe the current and historical position of a sample throughout the HT workflow. These metadata record information regarding the storage system and its internal position, as well as the sample’s location on transport lines or at workstation modules. Location metadata are essential for planning logistics within automated processes, enabling flow optimization and collision avoidance [180].

Synthesis-condition metadata constitute one of the most important categories of information assigned to a sample. They include process parameters such as chemical composition, temperature, pressure, step durations, reaction atmosphere, layer thickness, deposition rate, or processing conditions. In environments employing automated synthesis systems, these data are frequently recorded automatically and directly from control instruments. Synthesis metadata are critical for analyzing structure–property relationships and for training predictive models [4,171].

## 5. Challenges and Future Direction

Despite rapid advances in the automation of synthesis, processing, and characterization techniques, the development of fully autonomous high-throughput material discovery platforms continues to face a number of fundamental challenges. One of the main limitations is the inherent complexity and multiscale nature of material processes, which often involve highly nonlinear, coupled physicochemical phenomena. Processes such as sintering, additive manufacturing, and granulation are subject to interactions across multiple length and time scales, making real-time prediction and control difficult using conventional models. A major shortcoming is the lack of reliable in situ and in operando monitoring techniques that would allow for the recording of key material parameters during processing. Although significant progress has been made in sensor integration—such as optical, thermal, and spectroscopic methods—most systems still rely on indirect measurements or post-process characterization, which limits the implementation of true closed-loop control.

Another major challenge is data integration and standardization. HT processes generate extensive, heterogeneous datasets covering synthesis parameters, processing conditions, and characterization results. However, the lack of unified data structures, metadata standards, and interoperable platforms hinders the efficient exchange of data and the application of machine learning models across different systems and laboratories.

From an automation perspective, many material processing techniques remain only partially automated, and parameter adjustment, process stabilization, and decision-making still rely heavily on the knowledge and experience of operators. This dependency is particularly evident in powder processing, casting methods, and complex additive manufacturing techniques, where process outcomes are highly sensitive to even minor changes in parameters such as particle size distribution, rheology, temperature gradients, or energy input. As a result, empirical knowledge and a trial-and-error approach continue to dominate optimization strategies, especially in industrial settings where reliability takes precedence over experimental exploration. The limited ability to observe key process variables in real time forces operators to make indirect inferences about the system’s state, which introduces subjectivity and reduces the reproducibility of results. Strong nonlinearities and history-dependent behavior further amplify small disturbances, hindering deterministic control and necessitating continued reliance on expert supervision. Automation is also fragmented: although individual operations (e.g., dosing, temperature control, motion systems) may be highly automated, they are rarely integrated into a fully connected, closed-loop workflow. As a result, synthesis, processing, and characterization remain separate, preventing real-time optimization across the entire process chain. Achieving full autonomy will require adaptive, self-optimizing control systems that can continuously learn from process data and dynamically adjust parameters. Such systems must combine machine learning, physics-based models, and real-time sensing to enable predictive rather than reactive control. They will also need to be robust against disturbances, scalable across different material systems, and capable of operating under conditions of incomplete or uncertain data.

In the long term, the evolution of HT materials engineering is expected to be shaped by several key trends. First, the integration of artificial intelligence and machine learning with experimental platforms will enable the creation of “self-driving laboratories,” where the design, execution, and analysis of experiments will undergo iterative optimization. Second, the implementation of digital twins—virtual replicas of physical processes—will enable predictive modeling, process optimization, and real-time fault detection. Third, advances in multimodal sensing and sensor fusion are expected to significantly improve process observability, enabling more reliable closed-loop control.

At the same time, the development of modular and interoperable automation architectures will facilitate the integration of diverse synthesis and processing techniques within unified HT platforms. Efforts to standardize data formats, communication protocols, and experimental workflows will be essential for ensuring the scalability and reproducibility of results across different research environments. Ultimately, automation processes, advanced detection technologies, and data-driven methodologies are expected to transform materials engineering from a primarily empirical discipline into a predictive, autonomous, and digitally integrated field, meaningfully accelerating the discovery and optimization of next-generation materials.

Moreover, in civil, hydraulic, and geotechnical engineering, the integration of High-Throughput methods and computational automation enables comprehensive, large-scale evaluation of material performance beyond simple optimization. Automated data acquisition, modeling, and analysis allow efficient assessment of durability and behavior under complex service conditions. These approaches make it possible to account for multiple interacting factors such as permeability, temperature effects, and cyclic loading, using advanced data-driven models. As a result, HT and automation frameworks support the development of predictive tools for long-term stability and performance of materials and structures, demonstrating their applicability in civil engineering contexts [182,183,184].

## 6. Summary

This publication constitutes the first part of a comprehensive review dedicated to high-throughput (HT) synthesis and characterization methods in materials engineering, focusing on the hardware and logistical research architecture. The fundamental scientific problem addressed by the authors is the phenomenon of “data starvation,” which acts as a barrier to the implementation of artificial intelligence (AI) and machine learning (ML) algorithms in the design of advanced materials. The traditional experimental paradigm, dominated by manual procedures, is not only inefficient but also inherently flawed by systematic human bias, notably manifested in the non-publication of negative results. This phenomenon deprives predictive models of critical physicochemical boundary information regarding the design space, inevitably leading to the training of distorted models incapable of broad generalization. The proposed solution to this deficit is the implementation of fully automated HT platforms serving as objective “data factories,” which generate multidimensional, standardized, and machine-readable datasets while eliminating the influence of subjective human factors. The paper reviews the necessity of a paradigm shift from classical, discrete Design of Experiments (DoE) toward an innovative approach based on Continuous Gradient DoE. The realization of these objectives requires the application of advanced robotic infrastructure. The methods for automating liquid- and solid-phase precursor synthesis are comprehensively characterized, with highlighted use of the integrated flow stations and precise gravimetric systems. Particular attention is given to the engineering challenges associated with the automated processing of solid materials and composites. The review analyzes advancements in technologies such as smart polymer extrusion and injection molding with adaptive feedback control, inductive and arc melting of metals, modern powder metallurgy and sintering processes (including FAST/SPS techniques), as well as additive manufacturing (3D printing) monitored in real time via thermal and acoustic signals. The physical integration of individual synthesis, preparation, and characterization stations is achieved through the use of multi-axis robotic arms, precision manipulators with interchangeable end-effectors, and automated dispensing stations, which collectively determine the continuity and reproducibility of high-throughput experimental cycles. A critical element of the automated laboratory environment emphasized in the publication is the implementation of Continuous Material Management. Generating tens of thousands of material variants necessitates the use of advanced storage systems with diverse architectures—ranging from static and dynamic solutions to cold, belt-based, and drawer-based storage—serving as buffers and providing both short- and long-term sample retention. In parallel with physical logistics, informational logistics must also be comprehensively developed. Flawless tracking of the research workflow requires the application of physical sample tagging methods (such as 1D/2D barcodes, RFID, and NFC systems) or contactless computer vision tracking and color-coded marking. These technologies form the foundation for managing metadata in accordance with the FAIR principles (Findable, Accessible, Interoperable, Reusable), ensuring machine readability, unambiguous identification, and analytical consistency throughout the entire sample lifecycle. Summary of automated and high-throughput methods for materials synthesis and processing is presented in Table 14. Summarizing this part of the review, the main limitations to the development of fully autonomous laboratories are concluded, including the highly nonlinear dynamics of physicochemical processes, the lack of reliable in situ and in operando monitoring techniques, and the fragmentation of automation solutions. Consequently, the future of materials engineering will be shaped by the deepening integration of sensor data fusion, digital twins, and AI algorithms. Ultimately, this will enable the transition from reactive prediction to autonomous “self-driving laboratories” capable of optimizing the research process through a continuous, closed-loop feedback system.

## 7. Review Methodology

The literature reviewed in this work was collected using the Scopus and Google Scholar databases. The search process focused on publications related to high-throughput experimentation, laboratory automation, robotic materials processing, and data-driven materials engineering. The primary search keywords included: high-throughput materials, high-throughput experimentation, autonomous laboratory, self-driving laboratory, robotic synthesis, robotic materials processing, materials automation, automated materials discovery, combinatorial materials science, continuous gradient design of experiments, additive manufacturing automation, robotic additive manufacturing, automated characterization, sample management, sample tracking, RFID materials tracking, laboratory robotics, digital twins in materials science, machine learning for materials discovery, FAIR metadata, materials informatics, closed-loop optimization, and autonomous experimentation.

The search primarily covered publications from 2010 to 2025, although several earlier landmark studies were included when considered fundamental to the development of high-throughput materials research. More than 350 publications were initially identified through database searches and cross-referencing of cited literature. Following the removal of duplicates and the screening of titles, abstracts, and full texts for relevance to the scope of this review, 185 publications were selected for detailed analysis.

The inclusion criteria comprised peer-reviewed journal articles, review papers, and highly cited conference contributions addressing automation, high-throughput synthesis, materials processing, robotic systems, sample logistics, metadata management, and autonomous laboratory architectures. Publications focused exclusively on biological or pharmaceutical high-throughput systems without direct relevance to materials engineering were excluded. The final selection was used to provide a comprehensive overview of current technologies, challenges, and future directions in automated high-throughput materials research.

## Figures and Tables

**Table 1:** Comparison of selected review articles related to autonomous materials discovery and laboratory automation.

Review	AI/ML	Autonomous Experimentation	Robotics	Sample Management	Storage System
Tabor et al. [3]	✓	Partial	Partial	✗	✗
Pollice et al. [6]	✓	Partial	✗	✗	✗
Stach et al. [4]	✓	✓	✓	Partial	✗
Abolhasani & Kumacheva [5]	✓	✓	✓	Partial	✗
This review	✓	✓	✓	✓	✓

**Table 2:** Structure of the review series.

Part	Scope
Part I	HT synthesis, processing, robotics, sample logistics
Part II	HT characterization, sensors, feature extraction, multimodal data
Part III	AI, ML, digital twins, self-driving laboratories

**Table 3:** Overview of automated solution-phase synthesis platforms.

Platform	Type	Automation Features	Strengths	Limitation
Chemputer	batch	syringe pumps, valve networks	full reaction replication	limited scale
SynBot	batch	AI retrosynthesis + robotics	adaptive optimization	complex architecture
AutoSyn	flow	multi-stage flow network	high reproducibility, scalability	hardware complexity
AlphaFlow	flow + RL	reinforcement learning control	exploration of unknown space	computationally intensive

**Table 4:** Automation in melt-based processing.

Process	Control System	Monitoring	Key Limitation
Induction melting	PLC/SCADA	pyrometry	thermal gradients
Arc melting	ROS2 + sequencing	optical + current	unstable melt
Welding	AI + vision systems	camera + sensors	non-linear melt pool

**Table 5:** Automation in powder-based processing.

Process	Automation Level *	Monitoring	Key Challenge
Ball milling	medium-high	vibration, IoT	indirect measurement
Spray drying	medium	moisture sensors	particle size control
Wet granulation	high	CFD-DEM + digital twin	material turbulence
Dry granulation	high	PAT (NIR, imaging)	material variability

* automation level. Low—operator-dependent. Medium—partially automated. High—automated process with limited operator intervention. Very high—fully computer-controlled workflow.

**Table 6:** Overview of sintering technologies.

Process	Type	Automation Level *	Key Advantage	Limitation
FAST/SPS	electric	medium	fast densification	geometry modeling
laser sintering	optical	high	precision	defect control
UHS	Joule heating	high	ultra-fast	material constraints
hot pressing	thermo-mech	medium	dense materials	harsh environment

* automation level. Low—operator-dependent. Medium—partially automated. High—automated process with limited operator intervention. Very high—fully computer-controlled workflow.

**Table 7:** Automation in polymer molding processes.

Process	AI Control	Sensor Systems	Main Limitation
Extrusion	MPC + ANFIS	pressure/torque	nonlinear delay
Injection molding	neural nets	cavity sensors	material variability
Blow molding	reinforcement learning	IR heating control	drift

**Table 8:** Comparison of automation strategies in metal additive manufacturing.

Process	Monitoring Approach	AI/Control Role	Automation Level *	Key Limitation
LPBF/PBF	optical, thermal, acoustic, photodiodes	closed-loop energy control	high	chaotic melt-pool physics
DED/WAAM	cameras + robotic sensing	path planning + correction	high	geometric instability
general AM	digital twins	predictive defect correction	medium-high	data transferability

* automation level. Low—operator-dependent. Medium—partially automated. High—automated process with limited operator intervention. Very high—fully computer-controlled workflow.

**Table 9:** Comparison of automation strategies in polymer additive manufacturing.

Process	Monitoring Approach	AI/Control Role	Automation Level *	Key Limitation
FFF	vibration, IR, acoustic	parameter optimization	medium-high	anisotropy, clogging
SLA/DLP	exposure modeling	resin optimization	medium	post-processing bottleneck
SLS	thermal + ML sensing	defect detection	high	powder handling complexity

* automation level. Low—operator-dependent. Medium—partially automated. High—automated process with limited operator intervention. Very high—fully computer-controlled workflow.

**Table 10:** Key automation components in robotic laboratory workflows.

Component	Primary Function	Typical Systems	Key Advantage	Limitation
Robotic arms	sample transfer, module integration	linear rails, articulated robots	high throughput, flexibility	limited tactile feedback
Feeding stations	reagent/material dosing	loss-in-weight feeders, pipetting robots	high precision, reproducibility	clogging/calibration drift
Manipulators	task-specific operations (mixing, gripping, positioning)	modular end-effectors	task versatility, precision	task-specific constraints

**Table 11:** Comparison of storage systems in high-throughput laboratories.

Storage Type	Access Strategy	Automation Level *	Typical Use Case	Key Advantage	Main Limitation
Static storage	fixed location	low	reference/archive samples	simplicity, reliability	low flexibility
Dynamic storage	real-time relocation	high	adaptive HT workflows	space optimization	system complexity
Cold storage	temperature- controlled	medium	sensitive materials	thermal stability	energy demand
Belt-based storage	continuous flow	high	process integration	high throughput	poor random access
Drawer-based storage	modular retrieval	high	substrates, vials	balance of density and access	mechanical complexity

* automation level. Low—operator-dependent. Medium—partially automated. High—automated process with limited operator intervention. Very high—fully computer-controlled workflow.

**Table 12:** Comparison of automated storage systems in high-throughput laboratories.

System Type	Mobility Principle	Automation Level *	Typical Role in HT Workflow	Key Advantage	Main Limitation
AS/RS	robotic 3D positioning	very high	core storage + retrieval	full automation, high density	high complexity & cost
Flow-rack	gravity/roller flow	medium	buffer/intermediate storage	simplicity, fast access	limited flexibility
Shuttle-based	autonomous carts	high	scalable distributed storage	high scalability, random access	coordination complexity
Carousel-based	rotating storage units	medium-high	medium-capacity dynamic storage	fast access, compact design	limited capacity scaling

* automation level. Low—operator-dependent. Medium—partially automated. High—automated process with limited operator intervention. Very high—fully computer-controlled workflow.

**Table 13:** Comparison of sample identification and tracking technologies in automated laboratories.

Method	Principle	Line-of-Sight Required	Information Capacity	Automation Compatibility	Key Advantage	Main Limitation
Barcoding (1D/2D)	optical encoding	yes	medium–high	very high	low cost, simplicity	label damage, visibility constraint
RFID	wireless radio-frequency	no	high	very high	multi-tag reading, robustness	cost, metal/liquid interference
NFC	short-range RFID variant	very short	medium	medium-high	intuitive local interaction	very short range
Computer vision	image-based tracking	yes	variable	high	tag-free operation	no unique global ID
Color-coded tagging	visual markers	yes	low	high	simplicity, redundancy	low information density

**Table 14:** Summary of automated and high-throughput methods for materials synthesis and processing.

Method Class	Method	Material Class	Automation Level *	HT Potential **	Sensors	Main Limitation
Solution-Based Processing	Automated solution preparation and mixing	Chemicals/ Precursors	Very high	Very high	Gravimetric sensors, liquid level sensors, flow meters, conductivity, pH, temperature	Limited applicability for highly viscous or multiphase systems
	Sol–gel synthesis	Chemicals/ Precursors	High	High	Temperature, pH, viscosity, spectroscopy liquid handling sensors	Complex reaction kinetics and aging processes are difficult to control automatically
	Hydrothermal/solvothermal synthesis	Chemicals/ Precursors	Medium/High	Medium	Temperature, pressure, autoclave monitoring, conductivity	Batch-based operation and limited real-time observation
	Flow chemistry/continuous synthesis	Chemicals/ Precursors	Very high	Very high	Mass flow controllers, pressure sensors, temperature sensors, inline spectroscopy	Requires optimized reactor design and precursor compatibility
Melt -Based Processing	Induction melting	Metals and Alloys	High	Medium	Temperature, pyrometers, power monitoring	Limited compositional screening
	Arc melting	Metals and Alloys	Medium	Low	Current, voltage, temperature	Electrode wear, limited automation
	Welding	Metals and Alloys	High	Low/Medium	Vision, acoustic, thermal cameras	Weld-pool instability
Powders and Sintering	Sintering	Metals and Alloys	High	Medium	Temperature, pressure, atmosphere sensors	Long cycle times
	Spray drying	Ceramics	High	High	Moisture, temperature, pressure, airflow	Limited control of particle size distribution
	Wet granulation	Ceramics	Medium	Medium	Moisture, NIR, torque sensors	Complex rheology
	Dry granulation	Ceramics	High	Medium	Force, pressure, particle size sensors	Particle segregation
	Ball milling	Ceramics	Medium	Medium	Vibration, acoustic emission, temperature	Limited real-time monitoring
	Selective Laser Sintering	Ceramics	Medium	Medium	Thermal camera, pyrometer	Thermal gradients
	Ultra-High-Temperature Sintering	Ceramics	High	High	Current displacement, temperature	Limited sample size
	Hot Pressing	Ceramics	High	Medium	Temperature, pressure, displacement	Batch process
Thin films	Chemical Vapor Deposition	Metals and Alloys/Ceramics	High	High	Mass flow, pressure, OES, temperature	Complex chemistry
	Pulsed Laser Deposition	Ceramics	High	High	Laser energy, plume imaging, OES	Small deposition area
Molding	Extrusion	Polymers	Very high	High	Pressure, temperature, torque	Material-dependent rheology
	Injection Molding	Polymers	Very high	Very high	Pressure, temperature, cavity sensors	Mold cost
	Blow Molding	Polymers	Very high	High	Pressure, temperature	Limited geometries
Additive Manufacturing	Powder Bed Fusion	Metals and Alloys	High	High	Thermal camera, pyrometer, melt-pool monitoring	Residual stresses
	Direct Energy Deposition	Metals and Alloys	High	Medium	Vision, pyrometers, melt-pool sensors	Low dimensional accuracy
	Wire Arc Additive Manufacturing	Metals and Alloys	High	Medium	Thermal camera, arc sensors, vision	Surface quality
	Fused Filament Fabrication	Polymers	Very high	High	Temperature, machine vision	Interlayer defects
	Selective Laser Sintering	Polymers	High	High	Thermal camera, pyrometer	Powder handling
	Digital Light Processing	Polymers	Very high	Very high	Optical sensors, camera	Resin limitations
	Stereolithography	Polymers	High	High	Optical monitoring, camera	Post-processing requirements

* automation level. Low—operator-dependent. Medium—partially automated. High—automated process with limited operator intervention. Very high—fully computer-controlled workflow. ** HT potential. Low <10 samples/day. Medium 10–100 samples/day. High 100–1000 samples/day. Very high >1000 samples/day or continuous combinatorial processing.

## Data Availability

No new data were created or analyzed in this study. Data sharing is not applicable to this article.

## Abbreviations

The following abbreviations are used in this manuscript:

HT	Hight-Throughput
DoE	Design of Experiments
AI	Artificial Intelligence
PLC	Programmable Logic Controllers
SCADA	Supervisory Control And Data Acquisition
VAR	Vacuum Arc Remelting
FAST/SPS	Field Assisted Sintering Technology/Spark Plasma Sintering
DCS	Distributed Control Systems
CFD-DEM	Computational Fluid Dynamics—Discrete Element Method
PAT	Process Analysis Tools
NIR	Near-Infrared
SLS	Selective Laser Sintering
UHS	Ultra-High-Temperature Sintering
OES	Optical Emission Spectroscopy
CVD	Chemical Vapor Deposition
PLD	Pulsed Laser Deposition
ANFIS	Adaptive Neuro-Fuzzy Inference System
MPC	Model Predictive Control
HME	Hot Melt Extrusion
PID	Proportional-Integral-Derivative
V/P	Velocity-to-Pressure
CAE	Computer-Aided Engineering
AM	Additive Manufacturing
PBF	Powder Bed Fusion
DED	Direct Energy Deposition
LPBF	Laser Powder Bed Fusion
EBM	Electron Beam Melting
WAAM	Wire Arc Additive Manufacturing
FFF	Fused Filament Fabrication
SLA	Stereolithography
DLP	Digital Light Processing
APR	Adjustable Pallet Racking
NAPR	Narrow Aisle Pallet Racking
LHCS	Latent Heat Cold Storage
AGV	Automated Guided Vehicles
AS/RS	Automated Storage and Retrieval System
RFID	Radio Frequency Identification System
NFC	Near-Field Communication
UID	Unique Identifier
